# Molecular signature of extracellular matrix pathology in schizophrenia

**DOI:** 10.1111/ejn.15009

**Published:** 2020-11-13

**Authors:** Harry Pantazopoulos, Pavel Katsel, Vahram Haroutunian, Gabriele Chelini, Torsten Klengel, Sabina Berretta

**Affiliations:** ^1^ Department of Neurobiology and Anatomical Sciences University of Mississippi Medical Center Jackson MS USA; ^2^ Department of Psychiatry The Icahn School of Medicine at Mount Sinai New York NY USA; ^3^ Department of Neuroscience The Icahn School of Medicine at Mount Sinai New York NY USA; ^4^ Mental Illness Research Education Clinical Centers of Excellence (MIRECC) JJ Peters VA Medical Center Bronx NY USA; ^5^ Translational Neuroscience Laboratory Mclean Hospital Belmont MA USA; ^6^ Department of Psychiatry Harvard Medical School Boston MA USA; ^7^ Translational Molecular Genomics Laboratory Mclean Hospital Belmont MA USA; ^8^ Department of Psychiatry University Medical Center Göttingen Göttingen Germany; ^9^ Program in Neuroscience Harvard Medical School Boston MA USA

**Keywords:** extracellular matrix, glial cells, interneurons, microarray, neurodevelopment, perineuronal nets, schizophrenia, synaptic plasticity

## Abstract

Growing evidence points to a critical involvement of the extracellular matrix (ECM) in the pathophysiology of schizophrenia (SZ). Decreases of perineuronal nets (PNNs) and altered expression of chondroitin sulphate proteoglycans (CSPGs) in glial cells have been identified in several brain regions. GWAS data have identified several SZ vulnerability variants of genes encoding for ECM molecules. Given the potential relevance of ECM functions to the pathophysiology of this disorder, it is necessary to understand the extent of ECM changes across brain regions, their region‐ and sex‐specificity and which ECM components contribute to these changes. We tested the hypothesis that the expression of genes encoding for ECM molecules may be broadly disrupted in SZ across several cortical and subcortical brain regions and include key ECM components as well as factors such as ECM posttranslational modifications and regulator factors. Gene expression profiling of 14 neocortical brain regions, caudate, putamen and hippocampus from control subjects (*n* = 14/region) and subjects with SZ (*n* = 16/region) was conducted using Affymetrix microarray analysis. Analysis across brain regions revealed widespread dysregulation of ECM gene expression in cortical and subcortical brain regions in SZ, impacting several ECM functional key components. SRGN, CD44, ADAMTS1, ADAM10, BCAN, NCAN and SEMA4G showed some of the most robust changes. Region‐, sex‐ and age‐specific gene expression patterns and correlation with cognitive scores were also detected. Taken together, these findings contribute to emerging evidence for large‐scale ECM dysregulation in SZ and point to molecular pathways involved in PNN decreases, glial cell dysfunction and cognitive impairment in SZ.

## Graphical abstract

Growing evidence points to a critical involvement of the extracellular matrix (ECM) in the pathophysiology of schizophrenia (SZ). We tested the hypothesis that the expression of genes encoding for ECM molecules may be broadly disrupted in SZ across several cortical and subcortical brain regions. Gene expression profiling of 14 neocortical brain regions, caudate, putamen and hippocampus from control subjects and donors with SZ was conducted using Affymetrix microarray analysis. Analysis across brain regions revealed widespread dysregulation of ECM gene expression in cortical and subcortical brain regions in SZ. In (a) proportion of ECM‐related differentially expressed genes (DEG) in SZ by the brain regions. The superior temporal cortex (BA22) showed the highest number of DEGs. In (b) ECM‐related DEGs in SZ compared to UC across all of the analysed regions (blue‐downregulated; red‐upregulated). DEG's *t*‐score (*Y* axis) values plotted against fold change (*X* axis).

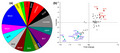

**Abbreviations**
ADAM: a disintegrin and matrix metalloproteinases
BA: Brodmann area
BCAN: brevican
CDR: Clinical Dementia Rating
CHST1: chondroitin/keratan‐6 sulfotransferase
CHSY1: chondroitin sulphate synthase 1
CNTF: ciliary neurotrophic factor
CSPG5/NGC: neuroglycan‐C
CSPGs: chondroitin sulphate proteoglycans
DEGs: differentially expressed genes
DSE: dermatan sulphate epimerase
DSPGs: dermatan sulphate proteoglycans
ECM: extracellular matrix
FGF: fibroblast growth factor
GALNS: galactosamine (N‐acetyl)‐6‐sulfatase
HAPLNs: hyaluronan and proteoglycan link proteins'
HSPGs: Heparan sulphate proteoglycans
IGF: insulin‐like growth factor
IGFBP3: insulin‐like growth factor‐binding protein 3
MMPs: matrix metalloproteinases
mRNA: Messenger RNA
NCAN: neurocan
OPCs: oligodendrocyte precursor cells
PMI: postmortem interval
PNNs: perineuronal nets
polySia‐NCAM: soluble polysialylated form of the neural cell adhesion molecule
RIN: RNA integrity number
SNP: single‐nucleotide polymorphism
SRGN: serglycin
SZ: Schizophrenia
TGF: transforming growth factor
UC: Unaffected control
VPA: valproic acid

## 1 INTRODUCTION

Several lines of evidence point to the involvement of the extracellular matrix (ECM) in the pathophysiology of schizophrenia (SZ). First, our group and others identified decreases of perineuronal nets (PNNs) in several brain regions in persons with SZ, including the amygdala, entorhinal cortex, prefrontal cortex, thalamic reticular nucleus and hippocampus (Enwright et al., 2016; Mauney et al., 2013; Pantazopolous et al., 2014; Pantazopoulos et al., 2010, 2015; Steullet et al., 2017). PNNs are organized perisynaptic ECM structures shown to be critically involved in regulating key aspects of synaptic transmission, including NMDA receptor trafficking, synaptic plasticity and protection from oxidative stress and functions with strong relevance to the pathophysiology of SZ (Beurdeley et al., 2012; Dityatev, Schachner, et al., 2010; Dityatev, Seidenbecher, et al., 2010; Frischknecht & Gundelfinger, 2012; Gogolla et al., 2009; Maeda et al., 2010; Pizzorusso et al., 2002). Formation of PNNs and maturation of the ECM contribute to the closure of critical periods of plasticity, conferring an adult form of restricted plasticity late in postnatal development (Dityatev & Rusakov, 2011; Fawcett, 2009; Gogolla et al., 2009; Gundelfinger et al., 2010; Nabel & Morishita, 2013; Pizzorusso, 2009; Pizzorusso et al., 2002), potentially overlapping with the prodromal syndrome of SZ. Our group has also shown abnormalities affecting recently discovered ECM structures, such as CS6 clusters (Pantazopoulos et al., 2015). Detectable by their predominant pattern of sulfation (CS6) on chondroitin sulphate chains, CS6 clusters were found to be markedly decreased in the amygdala of donors with SZ (Pantazopoulos et al., 2015). Studies in progress are beginning to uncover the role that CS6 clusters play in synaptic plasticity and regulation of glutamatergic transmission (Chelini et al., 2018; Hayashi et al., 2007; Horii‐Hayashi et al., 2010; Matuszko et al., 2017).

In addition to PNN and CS6 cluster deficits, marked alterations in expression of key ECM components, such as chondroitin sulphate proteoglycans (CSPGs), were detected in glial cells in persons with SZ (Pantazopoulos et al., 2010, 2015). The multifaceted functions of glia‐derived CSPGs are particularly relevant to the pathophysiology of this disorder, including myelination, dysregulation of nodes of Ranvier molecular and glial differentiation abnormalities (Bekku & Oohashi, 2010; Bekku et al., 2009, 2010; Frischknecht et al., 2014; Haroutunian et al., 2010; Katsel et al., 2008; Kerns et al., 2010; Mauney et al., 2015; Oohashi et al., 2002; Raabe et al., 2019; Roussos & Haroutunian, 2014; Roussos, Katsel, Davis, Bitsios, et al., 2012; Stedehouder & Kushner, 2017; de Vrij et al., 2019; Walker et al., 2018). Notably, evidence from gene expression studies suggests glial cell maturation deficits in SZ (Haroutunian et al., 2014). During development, glia‐derived CSPGs have been shown to regulate axon growth, axon guidance and axonal fasciculation (Hayashi et al., 2005; Ichijo, 2004; Klausmeyer et al., 2011; Kwok et al., 2012; Snow et al., 2003; Wilson & Snow, 2000). Importantly, during late postnatal development, CSPGs are key contributors to the powerful inhibition that myelination exerts on neurite outgrowth, thus contributing to the transition into a mature phase of restricted structural plasticity (Dours‐Zimmermann et al., 2009; Giger et al., 2010). In adulthood, CSPGs interact with oligodendrocyte precursor cells (OPCs), oligodendrocytes and myelinated axons. CSPGs, including the oligodendrocyte progenitor cell marker NG2, and the CSPG core protein brevican (BCAN), and neuroglycan‐C (also known as CSPG5), are involved in the regulation of glial progenitor cell maturation (Ichihara‐Tanaka et al., 2006; Kucharova & Stallcup, 2010; Lau et al., 2012; Pendleton et al., 2013; Siebert & Osterhout, 2011; Watanabe et al., 1995). Several CSPGs, for example, BCAN and NG2, are key components of the nodes of Ranvier, where they regulate axonal conduction (Bekku & Oohashi, 2010; Bekku et al., 2009; Dours‐Zimmermann et al., 2009; Huang et al., 2005; Hunanyan et al., 2010; Melendez‐Vasquez et al., 2005; Oohashi et al., 2002; Petrosyan et al., 2013). Of note, CSPGs potently regulate OPC differentiation and oligodendrocyte process outgrowth and myelination (Ichihara‐Tanaka et al., 2006; Kucharova & Stallcup, 2010; Lau et al., 2012; Pendleton et al., 2013; Siebert & Osterhout, 2011), and are strongly expressed by OPCs themselves (Bekku & Oohashi, 2010; Ichihara‐Tanaka et al., 2006; Stallcup & Huang, 2008; Trotter et al., 2010). BCAN is secreted by OPCs during active myelination (Bekku & Oohashi, 2010), while NG2 and neuroglycan‐C may be secreted, but also serve as cell surface receptors on OPCs (Ichihara‐Tanaka et al., 2006; Watanabe et al., 1995).

Genetic association studies and GWAS suggest that ECM abnormalities may reflect core genetic features underlying the pathophysiology of SZ (Muhleisen et al., 2012; Schizophrenia Working Group of the Psychiatric Genomics Consortium, 2014). These studies implicate several CSPGs, including neurocan (NCAN) and neuroglycan‐C and several matrix metalloproteinases (MMPs), such as MMP16, ‘a disintegrin and matrix metalloproteinases‐22’ (ADAM22), ADAMTSL3, ‘ADAMS with a thrombospondin domain 12’ (ADAMTS12) and ADAMTS‐16, (Bespalova et al., 2012; Dow et al., 2011; McGrath et al., 2013; Schizophrenia Working Group of the Psychiatric Genomics Consortium, 2014). A systematic gene expression analysis focused on ECM molecules across cortical and subcortical brain regions has not been carried out thus far. However, converging evidence point at widespread ECM gene expression abnormalities in several brain regions in persons with SZ (Fillman, Cloonan, Miller, et al., 2013; Katsel, Davis, Haroutunian, 2005a; Pantazopoulos et al., 2010; Pietersen et al., 2014). Taken together, the large number of genetic associations for ECM‐related genes and altered ECM gene expression in SZ indicate that PNN and CS6 cluster deficits, and altered expression of ECM molecules in glial cells may reflect downstream effects of multiple genetic factors.

In summary, compelling accumulating evidence points to a disruption of the ECM in SZ and to its relevance to the pathophysiology of this disorder. However, thus far, only a limited number of ECM molecules within few brain regions have been assessed. How pervasive is the disruption on the ECM in SZ and is there a region‐ and molecular‐specificity? As a first step in addressing these questions, we analysed the expression of genes encoding for ECM molecules in a dataset from 14 cortical and three subcortical brain regions from a cohort of control and SZ donors. We tested the hypothesis that ECM abnormalities are widespread in the brain, affecting a variety of cortical and subcortical brain regions. On the basis of emerging evidence for brain region‐specificity of ECM composition (Dauth et al., 2016), we postulated that region‐specific patterns of ECM abnormalities may occur in SZ. Finally, in light of the role the ECM plays in plasticity and learning, we hypothesized that ECM gene expression abnormalities may be associated with altered cognitive levels.

## 2 MATERIALS AND METHODS

Postmortem brains, donated by the next‐of‐kin of deceased subjects participating in studies of SZ, were received by the Mount Sinai NIH NeuroBioBank – ISMMS, Icahn School of Medicine at Mount Sinai. The subject cohort for these studies included unaffected (no evidence of neurological or neuropsychiatric diseases; see also below) control donors (UC; *n* = 22) and donors with SZ (*n* = 28; Table 1). Note that different numbers of subjects contributed to the different analysed regions (UC, *n* = 14 ± 3; SZ, *n* = 16 ± 4; Table 1). This is because some of the samples did not meet the RNA and hybridization quality control criteria adopted to ensure the validity of gene expression results for each sample analysed. All determinations of RNA and hybridization quality were made blind to diagnosis or brain region.

**Table 1:** Demographic and tissue preservation characteristics of the human HG‐U133AB (Affymetrix) microarray dataset and independent qPCR confirmation cohort

Characteristics	Microarray		qPCR (Hippocampus)	
	Normal controls	Schizophrenia	Normal controls	Schizophrenia
Total subjects	22	28	19	26
Subject # per region	14 ± 3^a^	16 ± 4		
Total samples/arrays	233	273		
Sex (M/F)	8/14	19/9	7/12	13/13
Age (years)	81.6 ± 11.6	74.5 ± 11.2	79.3 ± 3.7	72.8 ± 2.4
Brain pH	6.5 ± 0.2	6.4 ± 0.2	6.53 ± 0.05	6.47 ± 0.07
PMI (hr)	7.1 ± 4.6	12.9 ± 6.3	6.8 ± 1.5	11.4 ± 1.8

^a^ Data expressed as mean ± *SD*.

Table 1 describes the sample size, sex, age of death, postmortem interval (PMI) and pH of the samples. Differences in the age and tissue pH between the SZ and UC groups were not statistically significant (*p* > 0.1, two‐tailed Student's *t* test). Donors with SZ had significantly longer PMIs (*p* = 0.007, two‐tailed Student's *t* test); nevertheless, RNA integrity number (RIN) was high (RIN > 7) and equal between the comparison groups (*p* = 0.6, two‐tailed Student's *t* test). All donors died of natural causes.

Donors diagnosed with SZ had all been residents of Pilgrim Psychiatric Center (Long Island, NY) or associated nursing homes, and diagnosed in life with chronic intractable SZ (Dracheva et al., 2004; Powchik et al., 1998). Patients were diagnosed antemortem and assessed by a team of research clinicians according to Diagnostic and Statistical Manual of Mental Disorders, Fourth Edition (DSM‐IV) criteria. UC donors were community dwellers, residents of independent living facilities and nursing homes. On antemortem assessment (when available) and extensive medical chart review and caregiver interview, these donors did not show evidence of neurological or neuropsychiatric diseases and died of natural causes (myocardial infarction, congestive heart failure or non‐brain, non‐hepatic cancers (see Table 1) (Katsel, Davis, Gorman, et al., 2005). Upon extensive structured neuropathological assessment, specimens from SZ and control donors did not show evidence of any discernable neuropathology such as Alzheimer's disease, multi‐infarct dementia, etc. (Purohit et al., 1998). None of the SZ or control donors had an history of licit or illicit substance abuse (with the exception of tobacco use). All diagnostic assessment procedures were approved by the Icahn School of Medicine at Mount Sinai, Bronx, J.J. Peters VA Medical Center and Pilgrim Psychiatric Center IRBs, and postmortem consent for autopsy and research use of tissue was obtained from each next‐of‐kin or legally authorized official.

All frozen samples were dissected blind to diagnosis. Dissection and preparation of cortical samples were performed as described previously (Hakak et al., 2001). Briefly, for each anatomically identified cortical Brodmann area (BA) from the left hemisphere, a small block of tissue, measuring approximately 1.5 cm along the cortical surface, was dissected from 0.5‐ to 0.8‐cm thick coronal tissue sections. Each block included no more than a 1‐mm white matter ribbon below the deeper layer of the cortical block in order to ensure that the full extent of the layers was included while minimizing the amount of white matter. Regional dissections were based on anatomical landmarks as described in previous reports (Haroutunian et al., 2007; Katsel, Davis, Gorman, et al., 2005; Katsel, Davis, Haroutunian, 2005a).

Postmortem human samples were collected for each cohort subject from the following cortical areas and subcortical regions: prefrontal cortex (BA 8 [superior frontal gyrus], BA 10 [frontal pole], BA 44 [insular cortex], BA 46 [dorsolateral prefrontal cortex]), anterior cingulate gyrus (BA 24/32—at the level of the genu of the corpus callosum), posterior cingulate gyrus (BA 23/ 31—at the level of the pulvinar), parietal cortex (BA 7—inferior parietal lobule), temporal cortex (BA 20 [inferior temporal gyrus], BA21 [middle temporal gyrus], BA22 [superior temporal gyrus] and BA36/28 [parahippocampal gyrus/entorhinal cortex]) and occipital cortex (BA 17—primary visual cortex), hippocampus, caudate nucleus and putamen.

### 2.1 Assessment of Clinical Dementia Rating

The Clinical Dementia Rating (CDR) scale (Fillenbaum et al., 1996; Morris et al., 1993) was used to define the presence and severity of cognitive impairment for each case. As previously described (Byne et al., 2008), a multi‐step consensus approach was applied to the postmortem assignment of CDR scores based on cognitive and functional status during the last 6 months of life as described previously (Clinton et al., 2005; Haroutunian et al., 1999). Assignment of CDR included consideration of other measures of cognition, including longitudinally measured MMSE and neuropsychological test performance when available. The CDR assesses cognitive and functional impairments and provides specific severity criteria for classifying individuals as cognitively intact (CDR = 0) and mild cognitive impairment (CDR = 0.5–1), as well as moderate (CDR = 2) and severe (CDR = 3–4) dementia. UC group included only subjects with CDR scores of 0–1 in these studies. While CDR scores for SZ group were in the range of 0–4.

### 2.2 Postmortem samples for immunohistochemistry

Tissue blocks containing the whole amygdala from two normal control donors (Table 2) were obtained from the Harvard Brain Tissue Resource Center, McLean Hospital, Belmont, MA. Diagnoses were made by two psychiatrists on the basis of retrospective review of medical records and extensive questionnaires concerning social and medical history provided by family members. A neuropathologist examined several regions from each brain for a neuropathology report. The cohort for this study did not include subjects with evidence for gross and/or macroscopic brain changes, or clinical history consistent with cerebrovascular accident or other neurological disorders. Subjects with Braak and Braak stages III or higher were not included. Subjects had no significant history of substance dependence, other than nicotine and alcohol, within 10 years from death.

**Table 2:** Demographic and descriptive characteristics of the cohort used for immunohistochemical investigation samples for immunofluorescence studies

Case/age/sex	Cause of death/inflammation	brain weight (g)	PMI (hrs)	Hemisphere	Time of death
13/71/M	Cardiac arrest (A, N)	1,580	24.0	R	10:10
14/37/M	Electrocution (A, N)	1,460	18.75	R	21:00

Note

A: acute, no prolonged agonal period; C: chronic, with agonal period; I:infection/inflammatory condition present at time of death; N: no significant infection/inflammation present at time of death.

### 2.3 RNA isolation, microarray procedures, qPCR validation and statistical analysis

The subject cohort composition, demographic characteristics (Table 1) and the procedures for RNA isolation and the microarrays using Affymetrix (Santa Clara, CA) HG‐U133AB GeneChip set were as described previously (Haroutunian et al., 2006; Katsel, Davis, Gorman, et al., 2005; Katsel, Davis, Haroutunian, 2005b). RNA quality was assessed using a combination of a 260/280 ratio derived from the Agilent 2100 Bioanalyzer (Agilent Technologies, Palo Alto, CA). No correlation was observed between PMI and RNA quality, in line with published evidence (Dracheva et al., 2006; Katsel et al., 2013; Roussos, Katsel, Davis, Siever, et al., 2012; White et al., 2018). Microarrays were processed by Gene Logic Inc. (Gaithersburg, MD).

Affymetrix RMA microarray expression normalization and statistical comparisons were made using GeneSpring GX12 (Agilent Technologies, Santa Clara, CA) (Irizarry et al., 2003). Significantly different probe sets as defined by a Benjamini–Hochberg (Benjamini & Hochberg, 1995) adjusted moderated *t* test *p* < 0.05 were used for subsequent analyses. t‐scores were used as a standardized measure of gene expression change for each individual transcript across all of the analysed brain regions and described in detail previously (Katsel, Davis, Haroutunian, 2005b; Katsel et al., 2007, 2009; Smith et al., 2001). Contrast analysis is an extension of the fold change algorithm, which takes in account variability and estimates how well individual gene expression patterns fit a specified model (the contrast pattern vector). The contrast pattern vectors were set up in such a way as to permit detection of increased expression of genes in the tested sample set if they were present. Additional downstream statistical analyses were conducted with SPSS software (v.24) and employed correlational analysis, *t* tests, ANOVA and ANCOVA (when there were significant covariates).

### 2.4 Real‐time qPCR

The messenger RNA (mRNA) levels of four ECM‐related DEGs were measured by qPCR in a larger independent (from the microarray database) cohort (Table 1) using TaqMan probes and primer sets (Life Technologies, Carlsbad, CA). TaqMan probe identification numbers for selected genes and normalization controls are listed in Table S5. For relative quantification of mRNA expression, geometric means of the expression of three housekeeping genes (GUSB, RPLP0 and PPIA) were calculated using the standard curve method (Katsel et al., 2018). The housekeeping genes were selected for their stability after comparison of several different endogenous controls.

### 2.5 QRT‐PCR statistical analysis

We performed a logarithmic transformation of microarray raw intensities for DEGs to eliminate heterogeneity and used the transformed gene expression values for all statistical analyses. Another preliminary analysis assessed linear associations with sex, age, pH and PMI to evaluate their use as covariates. Sex, age, pH and PMI were used as covariates as indicated. Non‐parametric Mann–Whitney *U* test, or independent samples Kruskal–Wallis test were used as appropriate.

### 2.6 Immunocytochemistry

Free‐floating tissue sections were carried through antigen retrieval in citric acid buffer (0.1 M citric acid, 0.2 M Na_2_HPO_4_), heated to 80°C for 30 min and incubated in primary antibody rabbit anti‐SRGN (1:500, Sigma‐Aldrich, HPA000759) raised against synthetic human SRGN peptide corresponding to amino acids (MQKLLKCSRLVLALALILVLESSVQGYPTRRARYQWVRCNPDSNSANCLEEKGPMFELLPGESNKIPRLRTDLFPKTRIQDLNRIFPLSEDYSGSGFGSGSGSGSGSGSGFLTEMEQDYQLVDE) and subsequently in biotinylated secondary antibody goat anti‐rabbit IgG; 1:500; Vector Labs, Inc. Burlingame, CA, followed by streptavidin conjugated with horse‐radish peroxidase for 2 hr (1:5,000 μl; Zymed, San Francisco, CA), and, finally, in nickel‐enhanced diaminobenzidine/peroxidase reaction (0.02% diaminobenzidine, Sigma‐Aldrich, 0.08% nickel‐sulphate and 0.006% hydrogen peroxide in phosphate buffer). All solutions were made in PBS with 0.5% Triton X (phosphate buffer/saline‐Tx) unless otherwise specified.

Immunostained sections were mounted on gelatin‐coated glass slides, coverslipped and coded for blinded quantitative analysis. All sections included in the study were processed simultaneously within the same session to avoid procedural differences. Omission of the primary or secondary antibodies did not result in detectable signal. Validation information for the primary antibody for SRGN is provided by Sigma‐Aldrich as part of the Prestige antibodies collection at www.proteinatlas.org.

## 3 RESULTS

We analysed gene expression of 73 differentially expressed ECM genes (DEGs; *p*corr < 0.05) that matched GO terms: cell adhesion, cell–cell adhesion, cell‐matrix adhesion and hyaluronic acid binding across multiple brain regions in human postmortem brain samples of SZ and UC donors.

### 3.1 ECM expression across brain regions in UC donors

#### 3.1.1 Region‐specific expression of genes encoding for ECM molecules

**Figure 1:**
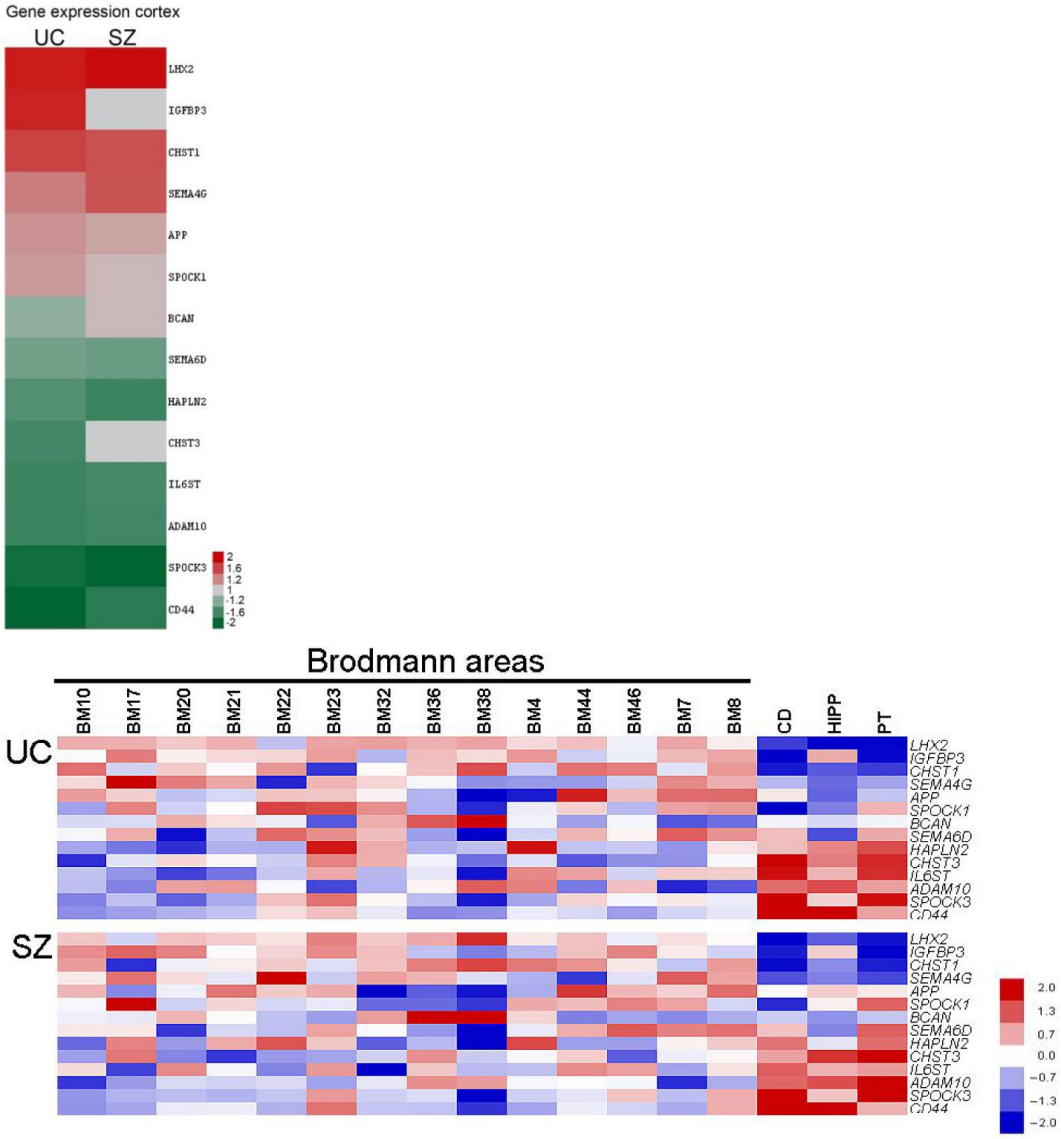
(a) ECM‐related transcription differences between cortical brain regions and subcortical areas are altered in SZ. A fold change scaled heatmap of the ECM‐related differentially expressed genes (DEGs) between cortical brain regions and subcortical regions in controls (UC) and in SZ. Shown the ratios of means of analyzed cortical regions versus subcortical regions (All *p* < 0.05 in UC). (b) Depicted are mean per area intensities of DEGs (rows) from the Brodmann areas (columns: as marked), caudate (CD), putamen (PT) and the hippocampus (HIPP). Data were standardized to have a mean of 0 and standard deviation 1 by linear transformation

ECM gene expression shows marked region‐specificity. A list of ECM DEGs demonstrating significant regional differences is shown in Figure 1, Table S1. Notably, highly significant differences were detected in comparisons between mRNA levels for DEGs in cortical versus subcortical regions, while differences between cortical regions were less pronounced. For instance, *BCAN*, *HAPLN2*, *SEMA6D* and *SPOCK3* showed significantly higher mRNA levels in subcortical regions compare to cortical areas. In contrast, the expression of *IGFBP3*, *LHX2*, *CHST1* and *SPOCK1* mRNA was lower in subcortical regions as compared to cortical areas (Figure 1, Table S1).

We also examined correlations between the variables (age, PMI and brain pH) and gene expression levels of DEGs. *CD44*, *IL6ST*, *CHST3*, *SRGN* and *DSE* showed very weak positive correlation (*r* > 0.2, *p* < 1E‐05, *n* = 228) with age across pooled cortical regions, while CHST1 showed significant negative correlation with age (*r* = −0.3, *p* = 5E‐06, *n* = 228). The effect of PMI was significant only for IL6ST (*r* = −0.24, *p* = 0.0003, *N* = 228). Effects of brain pH were significant for CHST3, EGFR and BCAN (*r* < −0.2, *p* < 0.001, *n* = 228).

### 3.2 Sex‐specific expression patterns

The expression of a number of ECM‐related genes was found to be significantly different in males versus females, with no interaction between sex and diagnosis. Specifically, the expression of syndecan 3, ADAM22 and amyloid β (A4) precursor protein was higher in males, while the expression of epidermal growth factor receptor was lower in males. In addition, the expression of CD44, dermatan sulphate epimerase (DSE) and ADAMTS1 was found to have a significant sex‐by‐diagnosis interaction (see below and Table S4).

### 3.3 Altered gene expression of ECM‐related molecules in schizophrenia: Region‐specificity

**Figure 2:**
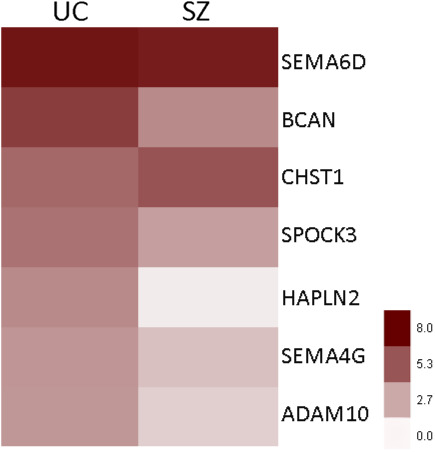
Interregional ECM‐related gene expression differences in cerebral cortex are diminished in SZ. An *F* value scaled heatmap of the ECM‐related DEGs in in cerebral cortex only in UC and SZ. One‐way ANOVA (All *p* < 0.01 in UC)

**Figure 3:**
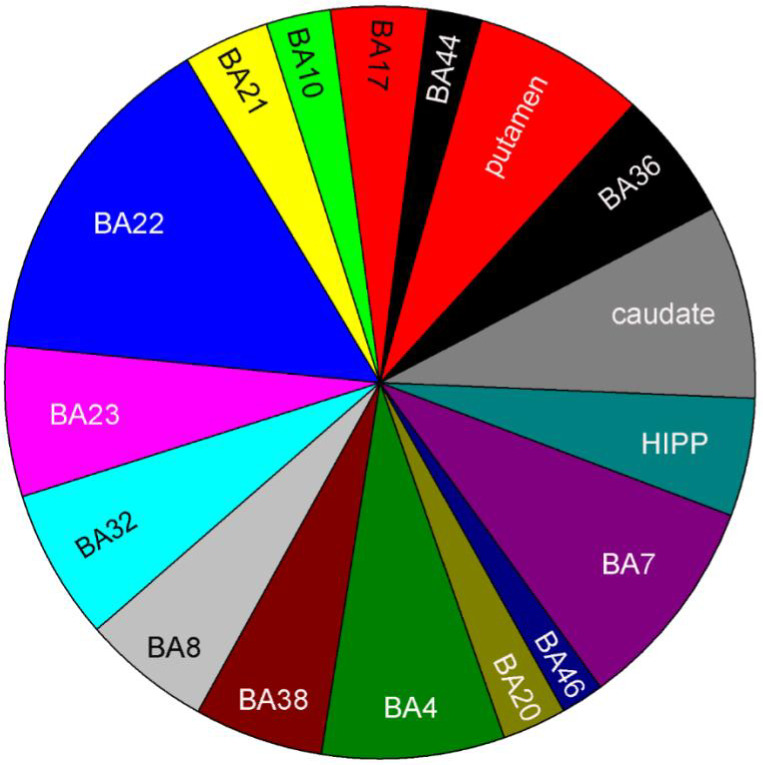
The superior temporal cortex (BA22) showed the most number of ECM‐related DEGs in SZ. Proportion of ECM‐related DEGs in SZ by the brain regions (Moderate *t*‐test; *p* < 0.001; FC(abs)>1.2). BA, Brodmann areas are as indicated; HIPP, hippocampus

Altered ECM‐related gene expression was detected in all 14 cortical and three subcortical brain regions examined (Figure 2, Table S1). Notably, robust regional variability of ECM gene expression in UC was found to be muted in donors with SZ, consistent with previous DEG findings in this disorder (Roussos, Katsel, Davis, Siever, et al., 2012) (Figure 2, Table S1). Proportional brain region changes for ECM‐related genes in SZ as compared to UCs are summarized in Figure 3. Superior temporal cortex (BA22) showed the highest number of ECM DEGs in SZ compared to UCs, again consistent with previous gene expression findings in SZ (Katsel, Davis, Gorman, et al., 2005). Parietal cortex (BA7), caudate, putamen, hippocampus, frontal area (BA4), part of precentral gyrus and cingulate cortices (BA23, 32) also showed high numbers of ECM DEGs.

### 3.4 Altered gene expression in schizophrenia impacts a broad range of ECM‐related molecules

**Figure 4:**
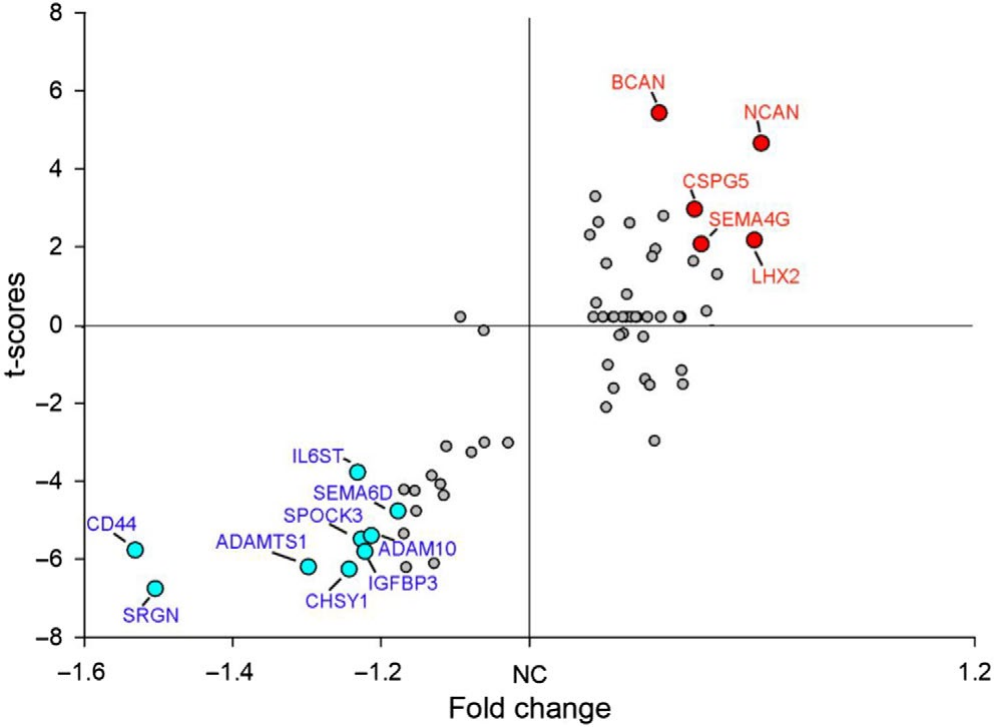
ECM‐related DEGs (Benjamini–Hochberg's FDR < 0.05) in SZ compare to UC across all of the analyzed regions (blue‐downregulated; red‐upregulated). DEG's *t*‐scores (*Y* axis) values plotted against fold change (*X* axis)

**Figure 5:**
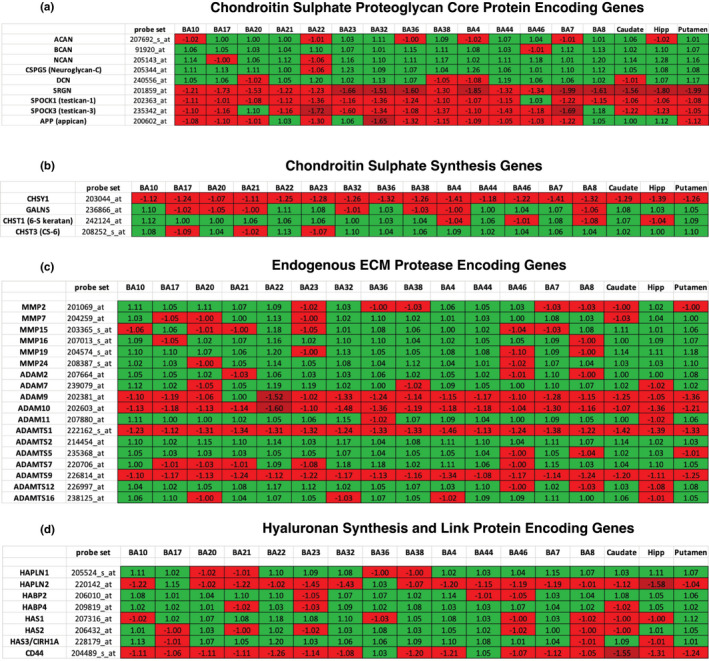
Differentially expressed ECM‐related genes in SZ across all brain regions examined. (a) Genes encoding chondroitin sulphate proteoglycan core proteins. (b) Chondroitin sulphate synthesis genes (c) Genes encoding endogenous ECM proteases (d) Genes involved in hyaluronan synthesis. Values represent fold change, probe set represents Affymetrix probe set used for each gene

**Figure 6:**
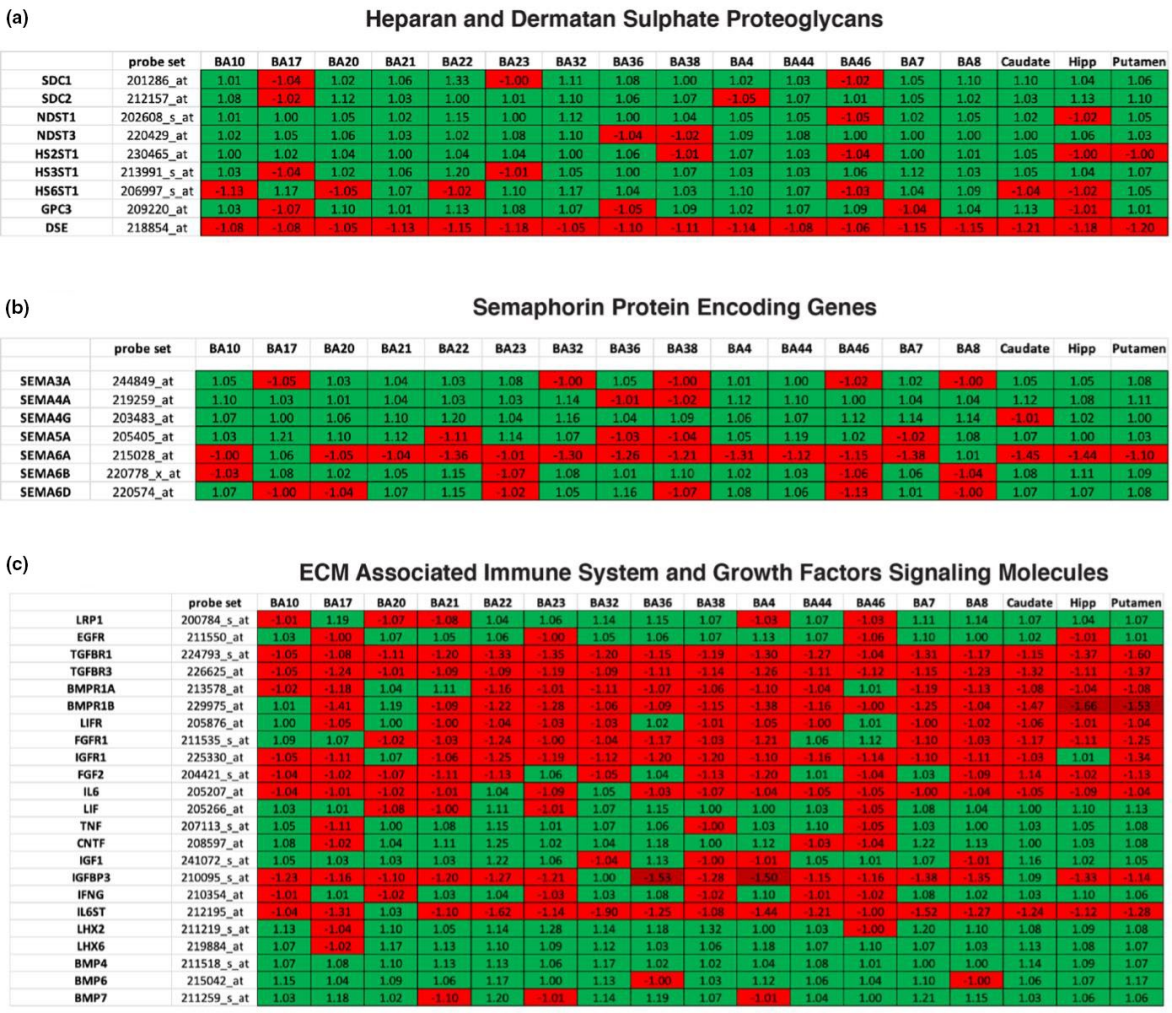
Differentially expressed ECM‐related genes in SZ across all brain regions examined. (a) Heparan and Dermatan sulphate proteoglycan core protein encoding genes. (b) Genes encoding semaphorin proteins (c) Immune system and growth factor molecules involved in ECM regulation. Values represent fold change, probe set represents Affymetrix probe set used for each gene

ECM DEGs in SZ include those encoding for key ECM components, such as CSPG core proteins, hyaluronan and link proteins, heparan and dermatan sulphate proteoglycan (DSPGs) and semaphorins, as well as enzymes involved in CS synthesis, factors regulating ECM posttranslational modifications, such as MMPs, and ECM regulation including immune system factors and growth factors (Figures 4, 5, 6).

#### 3.4.1 CSPGs

Dysregulation of genes encoding for CSPG core proteins in SZ was prominent and largely consistent across brain regions examined (Figure 5a). Upregulated gene expression was observed for ACAN (*p* < 0.05), BCAN, NCAN and neuroglycan‐C (*p* ≤ 0.001; see Figure 5a for fold change). Downregulated expression was detected for SRGN, SPOCK1, SPOCK3 and APP (*p* ≤ 0.001; see Figure 5a for fold change). The strongest fold changes overall were observed for SRGN (1.21–1.99 fold change; Figure 5a).

#### 3.4.2 Heparan sulphate proteoglycans (HSPGs) and dermatan sulphate proteoglycans (DSPGs)

Upregulated expression was observed for several HSPG genes across the several brain regions examined (*p* ≤ 0.001; see Figure 6a for fold change). Downregulated expression was detected for the gene encoding DSE (*p* ≤ 0.001; see Figure 6a for fold change).

#### 3.4.3 Hyaluronan‐related molecules and ‘hyaluronan and proteoglycan link proteins’ (HAPLNs)

Downregulated DEGs in SZ included HAPLN2 across most regions examined, and the hyaluronan receptor CD44 (*p* ≤ 0.001; see Figure 5d for fold change). In contrast, expression of genes encoding for HAPLN1 and several hyaluronan synthesis molecules and binding proteins was increased across most regions (*p* ≤ 0.001; see Figure 5d for fold change).

#### 3.4.4 Semaphorins

Several genes encoding for semaphorins, including SEMA4G, SEMA3A, SEMA6A and SEMA6D, were found to be dysregulated across most regions examined in SZ. Up‐ and downregulation were observed for each of these molecules in a region‐specific manner (*p* ≤ 0.001; see Figure 6b for fold change).

#### 3.4.5 CS synthesis molecules

DEGs in SZ across the brain regions tested included several enzymes involved in CS synthesis (Figure 5b). Expression of CHSY1 (chondroitin sulphate synthase 1), an enzyme member of the chondroitin N‐acetylgalactosaminyltransferase family which plays a critical role in the biosynthesis of chondroitin sulphate, was decreased across all brain regions examined (*p* ≤ 0.001; see Figure 5b for fold change). The expression of GALNS (galactosamine (*N*‐acetyl)‐6‐sulfatase), a *N*‐acetylgalactosamine‐6‐sulfatase required for the degradation of the glycosaminoglycans, keratan sulphate and chondroitin 6‐sulphate, was downregulated in selective BA regions (BA17, BA20, aBA21, BA38 and BA4), and upregulated in all other regions examined. Expression of the gene encoding the CS6 sulfotransferase CHST‐3 and the gene encoding the chondroitin/keratan‐6 sulfotransferase CHST1 were upregulated across most brain regions (*p* ≤ 0.001; see Figure 5b for fold change).

#### 3.4.6 MMPs

DEGs encoding for MMPs were consistently detected in SZ donors across brain regions tested (Figure 5c). Upregulated expression was detected for a large number of ADAM and ADAMTS genes, while ADAM9, ADAM10, ADAMTS1 and ADAMTS9 were downregulated across the majority of brain regions (Figure 5c). BA46 and the hippocampus showed higher numbers of downregulated ADAM and ADAMTS genes with respect to other brain regions (Figure 5c).

#### 3.4.7 Factors involved in ECM regulation

Expression of several genes encoding several growth factors known to regulate the ECM, including IGF1, BMP4,6,7 and CNTF, was found to be upregulated (*p* ≤ 0.001; see Figure 6c for fold change). Downregulated genes included those encoding for receptors for TGFβ, BMP, FGF and IGF across most brain regions (*p* ≤ 0.001; see Figure 6c for fold change).

### 3.5 Supporting evidence for ECM dysregulation from publicly available RNA‐seq data

In an effort to provide support for RNA microarray data in these studies, we cross‐referenced with data from existing, publicly available datasets from two RNA‐seq studies in SZ using Kaleidoscope (https://kalganem.shinyapps.io/BrainDatabases/, default settings) (Alganem et al., 2020; Hoffman et al., 2019; Wen et al., 2014). To this end, we selected a small number of the most downregulated and upregulated genes across brain regions in our data (i.e. those named in Figure 4, labelled in blue and red respectively) and tested whether they were found to be altered, in the same direction of change, in these two RNA‐seq datasets. Our findings show that of the 14 genes tested, nine showed genome‐wide significant changes in the same direction in at least one of the two datasets interrogated. Specifically, we found that CD44, ADAMTS1, ADAM10, SEMA6D and IL6ST were significantly downregulated in one or both of the RNA‐seq datasets, and BCAN, NCAN, CSPG5 and LHX2 were significantly upregulated (compare to Figure 4). Notably, ADAMTS1, which was significantly downregulated in our data and in both RNA‐seq datasets tested, also showed significant downregulation of the corresponding protein in the dataset by Wen et al. (Wen et al., 2014), providing strong support for a relevant role of these ECM molecules in SZ (Table S6).

### 3.6 Association of ECM gene expression with cognitive performance in unaffected control and SZ donors

**Figure 7:**
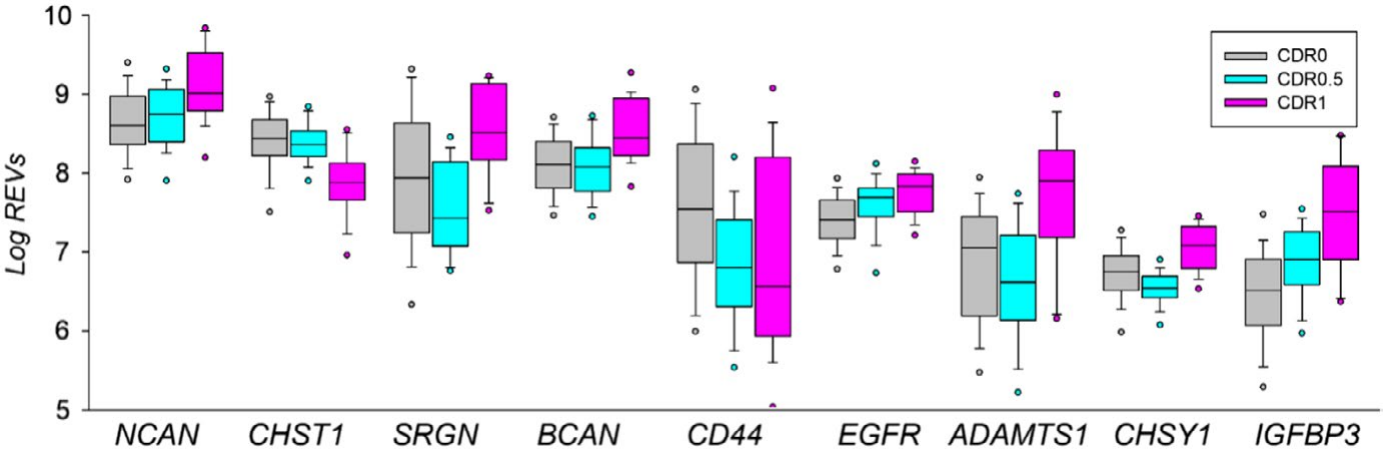
Significantly affected ECM‐related DEGs in persons with mild cognitive impairment (CDR 0.5–1) in UC. Box plot of log2 transformed raw expression values, REV (*p* < 0.001; Table S2)

In order to test the hypothesis that ECM gene expression levels may impact cognition, we assessed their correlation with CDR score in UC and SZ (Figure 7 and Table S2). In UC, several DEGs showed significant positive correlations (*r* > 0.3, *p* < 0.001) with clinical cognitive scores (CDRs 0.5–1), while CHST1 showed significant negative correlation with CDR (−0.335). We then tested the effect of CDR on gene expression changes of ECM DEGs in two UC subgroups, that is those with CDR = 0 versus those with CDR = 0.5–1. Across all brain regions tested, as well as across cortical regions only, 15 ECM genes showed significant differences between the two UC subgroups. These genes included those encoding for NCAN, SRGN, BCAN, CD44 and CHST1 (Figure 7 and Table S2).

In SZ donors, the correlations with CDR were positive for several genes, ADAMTS1, SPOCK1,3, although they were weaker than in UC < 0.3. The only negative correlation was for EGFR (−0.258) (Figure 7 and Tables S2 and S3). The CSPG/HSPG serglycin (SRGN), hyaluronan receptor CD44 and insulin‐like growth factor‐binding protein 3 (IGFBP3) were among the most consistently downregulated genes associated with both mild and severe cognitive impairment in subjects with SZ (Figure 7 and Tables S2 and S3).

### 3.7 SRGN is expressed on neurons in the human amygdala

**Figure 8:**
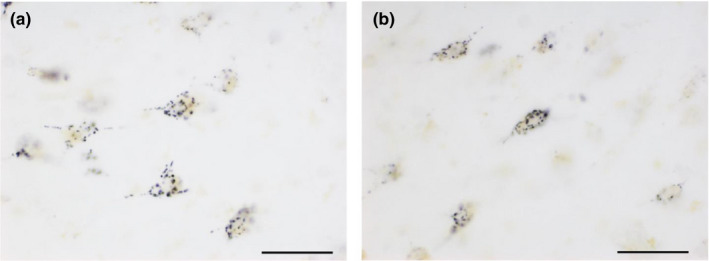
SRGN protein is expressed on pyramidal (a) and non‐pyramidal (b) neurons in the human amygdala. Scale bar = 100 μm

Immunohistochemistry on human amygdala samples from two UC subjects revealed widespread punctate SRGN labelling, located on both pyramidal and non‐pyramidal neurons in the amygdala (Figure 8). No labelling was observed on glial cells.

### 3.8 qPCR confirmation of gene expression changes in the hippocampus of subjects with SZ

**Figure 9:**
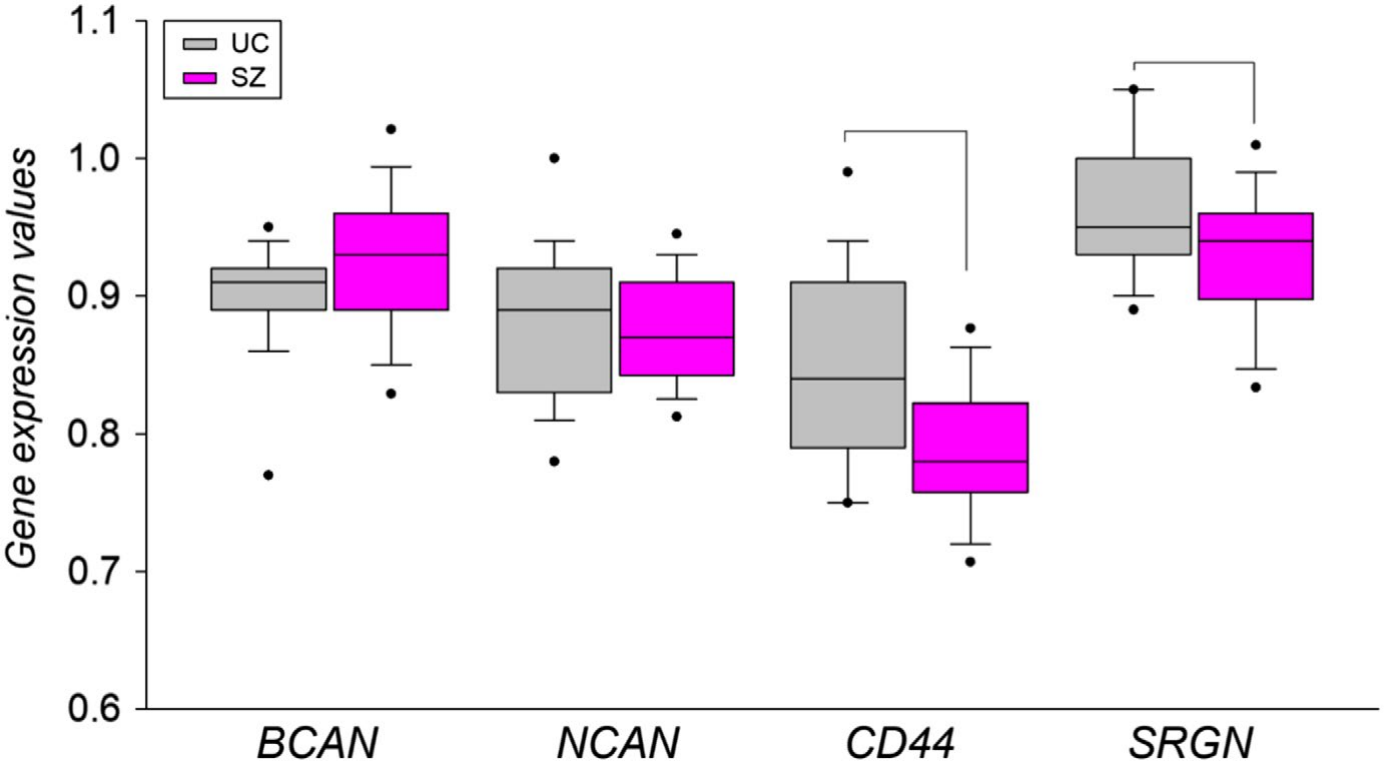
CD44 and SRGN gene expression changes in SZ were confirmed in hippocampus samples from a microarray‐independent cohort using qPCR. Box plot of normalized gene expression values. (ANCOVAs using age, sex, PMI and brain pH as covariates, *p* < 0.05)

Four of the top ECM‐related DEGs in our microarray analyses (BCAN, NCAN, CD44 and SRGN) were selected for confirmation qPCR analysis in the hippocampus of an independent cohort of cases with SZ and UC. As shown in Figure 9, mRNA expression of CD44 and SRGN was significantly reduced (*p* = 0.046 and *p* = 0.023, ANCOVAs controlled for age, sex, PMI and pH) in subjects with SZ. BCAN mRNA levels showed a trend towards increased expression in SZ that did not reach conventional significance level (*p* = 0.072, Mann–Whitney *U* test).

## 4 DISCUSSION

To our knowledge, these results represent the first large‐scale gene expression analysis focused on ECM molecules across multiple brain regions in persons with SZ. We report that the expression of ECM genes is altered across multiple cortical and subcortical brain regions in SZ and that these changes are associated with cognitive impairment. Notably, expression dysregulation in SZ impacts genes encoding for a broad range of ECM domains, from key ECM components, including several families of proteoglycans, hyaluronan receptors and link proteins, to factors involved in ECM synthesis, posttranslational modifications, such as sulfotransferases and MMPs, and immune system and growth factors involved in ECM regulation. Together, these findings point to a profound disruption of ECM‐related functions, likely to impact glial and synaptic functions, axonal conductance and myelination across cortical and subcortical circuits, in turn potentially disrupting neural connectivity, synaptic plasticity and, ultimately, cognition.

### 4.1 Technical considerations

We recognize several limitations, including lack of confirmation with QRT‐PCR and protein detection approaches to assess the relationship between mRNA and protein expression, and use of microarray technology, now largely replaced by RNA‐seq. We note that selected cohort groups included in these studies were matched by age at the time of death and tissue pH (indicator of agonal state). While PMI was significantly longer in SZ group, it did not affect the quality of RNA (RINs ≥ 7), consistent with large‐scale data published by others (White et al., 2018). Notably, no significant correlation between gene expression of ECM DEGs and PMI was detected in our studies.

While bulk and single‐cell RNA‐seq have become more common recently, data from targeted microarray studies and RNA‐seq have been reported to largely correlate with one another (Guo et al., 2013; Zhang et al., 2015). Although very limited in scope, comparisons between some of the gene expression changes across brain regions observed in our studies and two published RNA‐seq datasets (Hoffman et al., 2019; Wen et al., 2014) offer some degree of confirmation, with 9 of 14 genes selected showing significant changes at the genome‐wide scale and with the same direction of change (Table S6). These findings offer support for the validity of the microarray data reported in this present study. In addition, we conducted qPCR for the top 4 DEGs in our dataset using an independent cohort of hippocampus samples (Table 1), which provided partial confirmation of our findings (Figure 9). Notably, previous QRT‐PCR data from our group showed increased expression for BCAN, NCAN and ACAN in the amygdala of subjects with SZ (Pantazopoulos et al., 2010), providing additional confirmation for our microarray findings.

Another potential limitation is that it was not possible in this cohort to determine the effects of exposure to medications, such antipsychotics, valproic acid (VPA) and SSRIs. Thus, we cannot completely exclude the effect of exposure to pharmacological agents on gene expression of ECM‐related genes, which may have contributed to, or masked, some changes. Antipsychotics, lithium and VPA have all been reported to impact ECM molecules in rodent and human studies. In many cases, the effects of these drugs are opposite to changes observed in subjects with SZ, suggesting that these treatments may achieve therapeutic efficacy in part by correcting for changes in ECM molecules (Ruso‐Julve et al., 2019; Sainz et al., 2019; Yukawa et al., 2018). For example, expression of ADAMTS2, which was upregulated across all brain regions examined in our study, was increased in peripheral blood samples from subjects with SZ, and decreased following antipsychotic treatment (Ruso‐Julve et al., 2019; Sainz et al., 2019). Furthermore, antipsychotics were shown to inhibit dopamine‐induced expression of ADAMTS2 in neuronal cell culture, suggesting that antipsychotics partially correct for increased ADAMTS2 expression that we observed in subjects with SZ (Ruso‐Julve et al., 2019).

Similarly, lithium has been reported to correct some of the changes in gene expression observed in our study. Lithium was shown to downregulate the expression of NCAN, EGFR and MMPs (Rivera et al., 2019), molecules that were unregulated in subjects with SZ in our study (Figures 1 and 2). Lithium administration in rodents facilitated enzymatic CSPG digestion (Frederick et al., 2008; Yick et al., 2004) and alter expression of MMPs through its action as a GSK‐3β inhibitor (Hui et al., 2010; Kondratiuk et al., 2017; Tsai et al., 2010). However, chronic lithium treatment correlated with higher numbers of aggrecan and chondroitin 6‐sulphated PNNs in subjects with bipolar disorder in our previous human postmortem studies (Pantazopoulos et al., 2015), suggesting that lithium may maintain or protect PNNs. Studies on mice support a protective role of lithium on PNNs. Neonatal mice exposed to ethanol show reductions in PNNs, and treatment with lithium during ethanol exposure prevents PNN deficits (Lewin et al., 2018). VPA may also partially correct for changes in ECM molecules in SZ. VPA decreases the expression of MMPs, including MMP‐1, 2, 3, 9 and 13 in cancer studies (Artacho‐Cordón et al., 2015; Mitmaker et al., 2011; Yamanegi et al., 2012). This drug has also been reported to increase the mRNA expression of CD44 in cultured epithelial stem/progenitor cells (Masoud et al., 2019). In summary, although we cannot rule out the effects of exposure to psychotropic medications on our results, several reports in rodent and human studies strongly suggest that any potential effects are likely to mask or partially correct for, rather than induce, many of the changes in gene expression we observed in subjects with SZ.

### 4.2 Key ECM components are dysregulated in SZ

The ECM is a complex molecular network that surrounds all cells in the brain and forms specialized structures such as PNNs, CS6 clusters and perinodal aggregates surrounding the nodes of Ranvier (Bekku et al., 2009; Chelini et al., 2018; Dours‐Zimmermann et al., 2009; Hayashi et al., 2007; Horii‐Hayashi et al., 2010; Huang et al., 2005; Hunanyan et al., 2010; Matuszko et al., 2017; Melendez‐Vasquez et al., 2005; Oohashi et al., 2002, 2015; Pantazopoulos et al., 2015; Petrosyan et al., 2013). Although the representation of distinct members of its main molecular components is brain region‐specific (Dauth et al., 2016; Ueno et al., 2019) (see also our results above), the broad composition of the brain ECM includes hyaluronan, proteoglycans, link proteins (HAPLNs), glycoproteins, such as tenascins, and a large number of secreted molecular families, such a neurotrophins and semaphorins. In very general terms, the ECM supramolecular organization is based on hyaluronan, acting as the brain ECM backbone, anchored to cell membranes through molecules such as hyaluronan synthase and hyaluronan receptor CD44 and linked to CSPGs through HAPLNs, forming quaternary complexes (Carulli et al., 2010; Deepa et al., 2006; Kwok et al., 2010; Oohashi et al., 2015; Spicer et al., 2003; Yamaguchi, 2000). Tenascin R links CSPGs to each other (Lundell et al., 2004; Oohashi et al., 2015; Ruoslahti, 1996; Yamaguchi, 2000). Chondroitin sulphate chains, the sugar‐based components of CSPGs, also link a variety of protein factors (e.g. semaphorins, OTX2, neurotrophins) mediating their incorporation into complex ECM structures and availability to cell receptors (Fawcett, 2015; Fawcett et al., 2019).

Our results show that the expression of genes encoding for several ECM key components is disrupted in SZ. These include hyaluronan synthesis molecules, hyaluronan link proteins, the hyaluronan receptor CD44, CSPGs as well as HSPGs and DSPGs, and semaphorins. The potential implications of these findings are briefly discussed below.

#### 4.2.1 Hyaluronan‐related molecules

Our results (Figures 4 and 5d) show dysregulation of CD44, hyaluronan‐binding proteins HBP2 and HBP4 and hyaluronan synthases HAS1, HAS2 and HAS3/CIRH1A, and link proteins HAPLN1 and HAPLN2 in several brain regions in persons with SZ. Together, these changes may result in lower hyaluronan levels, deficits of hyaluronan anchoring to cell membranes and of its interactions with proteoglycans, overall contributing to a disruption of the ECM basic and supramolecular structure in SZ. The impact of these changes on brain functions may affect extracellular volume transmission, organization and stability of ECM structures such as PNNs, glial functions and synaptic remodelling (Bekku et al., 2003; Bijata et al., 2017; Carulli et al., 2006, 2010; Kochlamazashvili et al., 2010; Liu et al., 2004; Rauch, 2004).

Pervasive downregulation of CD44 mRNA (Figures 4 and 5d) is particularly notable. Altered CD44 expression has also been previously reported in the prefrontal cortex of subjects with SZ using RNA‐seq analysis (Fillman, Cloonan, Catts, et al., 2013; Fillman, Cloonan, Miller, et al., 2013), and in a human iPSC glial chimera mouse model of SZ (Windrem et al., 2017), where it was associated with glial cell maturation deficits. CD44 is a major hyaluronan receptor, connecting hyaluronan to the neuronal cytoskeleton and mediating its effects on synaptic plasticity (Bijata et al., 2017; Kochlamazashvili et al., 2010). While its extracellular N‐terminal binds hyaluronan, the cytoplasmic C‐terminal interacts with cytoskeleton‐associated proteins, including ankyrin, and modulates the activity of small Rho GTPases (Bourguignon, 2008; Carter & Wayner, 1988; Kalomiris & Bourguignon, 1988; Konopka et al., 2016; Roszkowska et al., 2016; Weber et al., 1996). CD44 was found to be associated with mature synapses, and reductions of its expression impacted excitatory neurotransmission and dendritic spine shape and enlargement following neuronal stimulation, effects mediated by actin cytoskeleton (Roszkowska et al., 2016). Elegant studies by Bijata and coworkers identified CD44 as a substrate for MMP9, showing that stimulation‐driven increases of MMP9 lead to CD44 cleavage and in turn to dendritic spine structural and electrophysiological changes (Bijata et al., 2017). Finally, CD44 has been shown to play a role in promoting astrocyte maturation (Liu et al., 2004), suggesting a potential contribution to glial progenitor maturation deficits in SZ (Magistri et al., 2016; Windrem et al., 2017).

Also notable in our results is the dysregulated expression of HAPLN1 and HAPLN2 mRNA in SZ (Figure 5d). HAPLN1 mRNA was upregulated in the majority of brain regions tested, while gene expression for HAPLN2 was downregulated across most regions (Figure 5d). Altered HAPLN1 expression in SZ was also reported in a previous gene expression analysis study of the superior temporal gyrus (Pietersen et al., 2014). HAPLN1 and HAPNL2 are expressed by neurons associated with PNNs (Bekku et al., 2003; Carulli et al., 2006; Rauch, 2004). Mice lacking HAPLN1 show attenuated PNNs and maintain the juvenile form of synaptic plasticity (Carulli et al., 2010), whereas mice lacking HAPLN2 show attenuated PNNs and BCAN expression (Bekku et al., 2012), suggesting that HAPLN1 and HAPLN2 are differentially associated with specific subtypes of PNNs. HAPLN2 has also been shown to represent a constitutive part of the ECM surrounding the nodes of Ranvier (Bekku et al., 2010; Oohashi et al., 2002). Decreased HAPLN2 mRNA expression in SZ suggests a disruption of axonal saltatory conduction, potentially contributing to altered connectivity in this disorder (Canu et al., 2015; Roussos & Haroutunian, 2014; Schmitt et al., 2011; Walterfang et al., 2006).

#### 4.2.2 Proteoglycans

CSPGs are major components of the brain ECM. Thought to be the ‘organizers’ of the ECM (Yamaguchi, 2000), CSPGs are key functional elements of PNNs, perinodal ECM and CS6 clusters (Bandtlow & Zimmermann, 2000; Bekku & Oohashi, 2010; Bekku et al., 2009; Carulli et al., 2005, 2006; Chelini et al., 2018; Dours‐Zimmermann et al., 2009; Fawcett et al., 2019; Hartig et al., 1994; Hayashi et al., 2007; Horii‐Hayashi et al., 2008, 2010; Hunanyan et al., 2010; Kwok et al., 2011; Maeda et al., 2010; Matthews et al., 2002; Matuszko et al., 2017; Melendez‐Vasquez et al., 2005; Murakami et al., 1999; Murakami & Ohtsuka, 2003; Pantazopoulos et al., 2015; Petrosyan et al., 2013; Seidenbecher et al., 2002; Vitellaro‐Zuccarello et al., 2001). Among the brain CSPGs, ACAN, BCAN, VCAN and NCAN prominently contribute to the composition of PNNs, regulating their functions (Deepa et al., 2006; Giamanco & Matthews, 2012; Giamanco et al., 2010; Suttkus et al., 2014). These CSPGs also contribute to various degrees to the composition of perinodal ECM (Bekku & Oohashi, 2010; Bekku et al., 2009; Dours‐Zimmermann et al., 2009; Hunanyan et al., 2010; Melendez‐Vasquez et al., 2005; Petrosyan et al., 2013). In addition, several CSPGs, including BCAN, NG2 and CSPG5, are expressed by glial cells and potently regulate OPC differentiation, oligodendrocyte process outgrowth and myelination (Ichihara‐Tanaka et al., 2006; Kucharova & Stallcup, 2010; Lau et al., 2012; Pendleton et al., 2013; Siebert & Osterhout, 2011).

Several genes encoding for CSPG core proteins were upregulated across most cortical regions examined, including NCAN, BCAN and CSPG5 (Figures 4 and 5a, Table S2). We previously reported increased expression of several of these CSPG genes in the amygdala of subjects with SZ, including ACAN, BCAN and NCAN (Pantazopoulos et al., 2010). GWAS data in SZ suggest that risk gene variants may contribute to some of these expression changes. For instance, association with SZ of the single‐nucleotide polymorphism (SNP) rs1064395 in the NCAN gene has been robustly demonstrated (Cichon et al., 2011; Muhleisen et al., 2012; Schultz et al., 2014; Wang et al., 2016, 2018). This SNP has also been associated with cognitive dysfunction in SZ (Wang et al., 2016) as well as in unaffected subjects (Raum et al., 2015). NCAN expression was reported to be significantly increased in the frontal cortex of carriers of the rs1064395 vulnerable allele (Wang et al., 2016). Consistent with these findings, NCAN expression was broadly increased in our study, and associated with cognitive impairment as indicated by CDR scores (Figure 7, Tables S2 and S3).

BCAN is expressed by immature oligodendrocytes during the pro‐myelinating developmental stage, and expression of BCAN is absent once these cells develop into mature oligodendrocytes (Ogawa et al., 2001). Increased expression of BCAN across most regions examined (Figures 4 and 5a) may impact the maturation process of these cells, perhaps maintaining them in an immature state. In addition, neuroglycan‐C (CSPG5) was also upregulated across most regions examined (Figure 5a). Neuroglycan‐C has been implicated in GWAS studies of SZ (Schizophrenia Working Group of the Psychiatric Genomics Consortium, 2014), and is involved in promoting elongation of OPC processes (Ichihara‐Tanaka et al., 2006). Altered neuroglycan‐C expression associated with glial cell maturation deficits was reported in human iPSC glial chimera mouse model of SZ (Windrem et al., 2017).

In contrast, genes encoding for CSPG/HSPG core proteins SRGN, SPOCK1 and SPOCK3 were downregulated across most regions (Figures 4 and 5a). Altered SPOCK1 expression was reported in the same cohort included in our previous gene expression profiling study (Katsel, Davis, Haroutunian, 2005a) and altered SPOCK3 expression was reported in a previous gene expression study of the superior temporal gyrus (Pietersen et al., 2014). Notably, SRGN was the most consistently downregulated gene across all cortical regions examined (Figures 4 and 5a) and one of the genes strongly associated with cognitive impairment in subjects with SZ (Figure 7, Tables S3 and S4). Consistent with this finding, decreased SRGN expression has been reported in olfactory epithelium biopsy samples from subjects with SZ in association with visual learning and memory performance (Horiuchi et al., 2016). The expression of SRGN in the brain has not been well characterized, although it has been reported to increase in experimental autoimmune encephalomyelitis in mice and in glioma (Roy et al., 2017; Warford et al., 2018). In human amygdala, we found that SRGN protein product is observed as a distinctly punctate labelling within the neuron cytoplasm (Figure 8), suggesting a vesicular localization potentially consistent with SRGN expression in mast cells and hematopoietic cells, where it is located in cytoplasmic secretory granules, and binds to several cytokines and growth factors (Korpetinou et al., 2014; Sarrazin et al., 2011). SPOCK1 and SPOCK3 encode for CSPG/HSPGs also referred to as testicans, shown in the mouse brain to be expressed in the postsynaptic domain of pyramidal neurons (Bonnet et al., 1996), and in the human brain in cortical and subcortical neurons (Marr et al., 2000). A role for SPOCK1 (testican‐1) in neurodevelopment, adult brain functions and neuropathology is suggested by emerging literature (Barrera‐Ocampo et al., 2016; Bonnet et al., 1996; Chen et al., 2017; Dhamija et al., 2014; Jabbari et al., 2019).

#### 4.2.3 Semaphorins

Semaphorins, and their receptor plexins, play a key role in axonal guidance, synaptic pruning, regulation of dendritic processes and have been proposed to contribute to the stabilization of synaptic transmission in the developing and mature brain (Alto & Terman, 2017; Carcea et al., 2014; Gu et al., 2019; Koncina et al., 2007; Lett et al., 2009; O'Connor et al., 2009; Orr et al., 2017; Pasterkamp & Giger, 2009; Simonetti et al., 2019). Many semaphorins are secreted into the ECM, where they interact with CSPGs and HSPGs and are incorporated into PNNs (Dick et al., 2013; Fawcett, 2015; Kantor et al., 2004; Yuzaki, 2018). Not surprisingly given their functions, semaphorins have being implicated in a growing number of neurodevelopmental, psychiatric and neurodegenerative disorders (Fawcett, 2015; Gil & Del Río, 2019; Gutiérrez‐Franco et al., 2017; Lee et al., 2019; Pasterkamp & Giger, 2009; Roth et al., 2009; Schott et al., 2016; Smith et al., 2015; Yang et al., 2017).

Our results show the dysregulation of the expression of several genes encoding for semaphorins in people with SZ (Figures 4 and 6b). Dysregulation of SEMA6D expression in our data is consistent with recent GWAS data reporting a risk variant in SZ (Wang et al., 2018). Downregulated semaphorin expression, including SEMA6D, has been shown in the prefrontal cortex of people with SZ (Arion et al., 2010). SEMA6A was consistently downregulated across most brain regions (Figure 6b). A potential contribution of SEMA6A to SZ is suggested by data showing that mice deficient in SEMA6A display neurodevelopmental, behavioural, and electrophysiological deficits, including altered connectivity of limbic and cortical regions, changes in EEG activity and social and exploratory behaviours, which are alleviated by the antipsychotic chlorpromazine (Runker et al., 2011). SEMA3A was upregulated in SZ across most regions examined (Figure 6b). Similar findings were reported in the prefrontal cortex and in the cerebellum of subjects with SZ, associated in the latter region with decreased expression of synaptic genes (Eastwood et al., 2003; Gilabert‐Juan et al., 2015). Notably, SEMA3A is a PNN component, accumulating within these ECM structures during the closure of critical periods of development (Carulli et al., 2013; Dick et al., 2013; Fawcett et al., 2019; Vo et al., 2013; de Winter et al., 2016). Its decreased expression across several brain regions may contribute to PNN deficits in SZ (Mauney et al., 2013; Pantazopolous et al., 2014; Pantazopoulos et al., 2010, 2015).

### 4.3 Molecular factors involved in ECM synthesis, posttranslational modification and regulation are disrupted in SZ

#### 4.3.1 CS synthesis molecules

Genes encoding for CSPG synthesis molecules were downregulated across most regions examined (Figure 5b). Chondroitin sulphate synthase 1 (CHSY1), a key enzyme in the biosynthesis of chondroitin sulphate chains (Kitagawa et al., 2001), and GALNS, encoding a lysosomal exohydrolase involved in the degradation of keratan and chondroitin 6‐sulphate at the non‐reducing end of CS and KS chains (Di Ferrante et al., 1978), were robustly downregulated across several regions (Figures 4 and 5b). The genes encoding for the CS6 and KS6 sulfotransferases (CHST3 and CHST1) were upregulated in most regions examined (Figure 5b). The involvement of many of these enzymes in CS6 and KS6 sulfation is particularly relevant to findings from our group showing reductions in SZ of CS6 clusters, ECM formations potentially contributing to the modulation of synaptic plasticity (Chelini et al., 2018; Hayashi et al., 2007; Horii‐Hayashi et al., 2010; Matuszko et al., 2017).

#### 4.3.2 ECM remodelling enzymes

Our results show that a large number of ECM remodelling enzymes are dysregulated in SZ (Figure 5c). This broad class of secreted proteases, which include MMPs, ADAMS and ADAMTS, may be particularly relevant to the pathogenesis of SZ given their role in dynamically regulating ECM neurochemical properties in response to neuronal activity (Abdolmaleky et al., 2005; Hobohm et al., 2005; Malemud, 2006; Medina‐Flores et al., 2004; Muir et al., 2002; Rivera et al., 2010). For instance, MMPs are integral components of PNNs, affect excitatory transmission and interact with integrins to mediate spine volume changes induced by LTP and LTD (Nagy et al., 2006; Rossier et al., 2015; Wang et al., 2008). Among the several ECM remodelling enzymes found to be dysregulated in SZ in our study (Figure 5c), we briefly mention MMP16, ADAMTS1 and ADAM 10. Increased gene expression of MMP16 was observed across most regions examined (Figure 5c). GWAS data indicate that MMP16 may represent a vulnerability gene for SZ (Ma et al., 2018; Schizophrenia Working Group of the Psychiatric Genomics Consortium, 2014), and increased IgG levels against MMP16 fragments were reported in plasma samples of people with SZ (Whelan et al., 2018). Furthermore, altered MMP16 gene expression in SZ was also reported in a gene expression profiling study of the superior temporal gyrus (Pietersen et al., 2014). While the role of this MMP in the brain has not been explored to date, it may represent a relevant target for future investigations.

ADAMTS1 and ADAM10 mRNAs were found to be robustly decreased across all brain regions in our study (Figures 4 and 5c), an expression change supported at genome‐wide significance by independent RNA‐seq datasets (Hoffman et al., 2019; Wen et al., 2014) (Table S6). Notably, these genes have been identified as genome‐wide risk loci for Alzheimer's disease and are dysregulated in a number of brain disorders (Kunkle et al., 2019; Miguel et al., 2005), suggesting a broad involvement in brain functions and disease. Particularly relevant to the pathophysiology of SZ and other psychiatric disorders are findings showing that ADAM10 is involved in the regulation of glutamatergic NMDA transmission in prefrontal cortex GABAergic neurons, specifically affecting the balance of synaptic/extrasynaptic NMDAR‐mediated signalling through the regulation of soluble polysialylated form of the neural cell adhesion molecule (polySia‐NCAM) (Varbanov & Dityatev, 2017). ADAM10 may thus represent a key player in mechanisms affecting regulation of excitatory transmission within prefrontal cortex intrinsic circuits (Beneyto et al., 2007; Beneyto & Meador‐Woodruff, 2008; Eastwood & Harrison, 2006; Glausier et al., 2014; Glausier & Lewis, 2017; Gonzalez‐Burgos & Lewis, 2012; Lewis et al., 2005; Nacher et al., 2013; Penzes et al., 2013; Shan et al., 2012; Sweet et al., 2010; Vicente et al., 1997; Weickert et al., 2013). Additional support for a role of ADAM10 in neural transmission and plasticity comes from evidence that it mediates neuron‐to‐microglia signalling as a key mechanism for cortical synaptic remodelling (Gunner et al., 2019). ADAMTS1 has also been shown to be involved in synaptic plasticity, potentially through cleavage of CSGPs such as BCAN (Howell et al., 2012; Yuan et al., 2002). Notably, mice lacking ADAMTS1 display sex‐specific synaptic deficits, with decreases of cortical synaptic protein levels specifically in female mice (Howell et al., 2012). This report resonates with our findings of sex by diagnosis interaction of decreased expression of ADAMTS1 in SZ (Table S4), suggesting that decreased ADAMTS1 may contribute to synaptic deficits in a sex‐dependent manner in this disorder.

#### 4.3.3 Immune system and growth factor molecules involved in ECM regulation

Overwhelming evidence implicates signalling pathways involved in immune/inflammatory responses and neurotrophic functions in the pathophysiology of a growing number of brain disorders including SZ (Alam et al., 2017; Bauer & Teixeira, 2019; Haroon et al., 2017; Heneka et al., 2015; Heppner et al., 2015; Hori & Kim, 2019; Khandaker et al., 2015; Leffa et al., 2018; McGeer et al., 2016; Michopoulos et al., 2017; Miller et al., 2018; Mondelli et al., 2017; Mongan et al., 2019; Müller, 2018; Najjar et al., 2013; Newcombe et al., 2018; Prata et al., 2017; Réus et al., 2015; Skaper et al., 2018; Sochocka et al., 2017; Zhang et al., 2016). A large number of these signalling pathways, included in these analyses, are intricately associated with ECM regulation (Beroun et al., 2019; Berretta et al., 2015; Colton, 2009; De Luca et al., 2020; Galea & Perry, 2018; Gaudet & Popovich, 2014; Haylock‐Jacobs et al., 2011; Smolders et al., 2019; Sobel, 2001; Stephenson & Yong, 2018; Wiemann et al., 2019; Zeis et al., 2016). It may thus not be surprising that the expression of many elements of immune/neurotrophic signalling pathways was found to be dysregulated in our study (Figure 6c). For example, gene expression of several members of the transforming growth factor (TGF), fibroblast growth factor (FGF), ciliary neurotrophic factor (CNTF) and insulin‐like growth factor (IGF) signalling pathways was found to be altered in SZ (Figure 6c). Interactions between these signalling pathways and ECM molecular factors are well documented and provide a coherent body of evidence for their role in regulating glial development and maturations as well as glial functions in the mature brain (Baghdassarian et al., 1993; Chesik et al., 2008; Galvin et al., 2010; Garwood et al., 2004; Lillien et al., 1990; Mayer et al., 1994; Milner & Campbell, 2003; Müller et al., 1995; Toru‐Delbauffe et al., 1992). Several CSPGs, including BCAN, NG2 and CSPG5, are expressed by glial cells under immune/neurotrophic factor regulation and in turn potently regulate OPC differentiation and oligodendrocyte process outgrowth and myelination (Bekku et al., 2010; Ichihara‐Tanaka et al., 2006; Kucharova & Stallcup, 2010; Lau et al., 2012; Pendleton et al., 2013; Siebert & Osterhout, 2011; Stallcup & Huang, 2008; Trotter et al., 2010). Furthermore, several of these genes, including BCAN, CSPG5, CD44 and BMP signalling molecules, have been implicated in the human iPSC glial chimera mouse model of SZ and associated with altered glial cell maturation in cells derived from subjects with SZ (Liu et al., 2019; Windrem et al., 2017). Together, these findings suggest that disrupted interactions between immune/neurotrophic factors and the ECM may contribute to glial abnormalities in SZ (Dietz et al., 2020; Haroutunian et al., 2014; Laskaris et al., 2016; Mallya & Deutch, 2018; Najjar & Pearlman, 2015; Stedehouder & Kushner, 2017).

## 5 CONCLUSIONS

Our findings point to extensive dysregulation of ECM‐related genes in SZ, impacting multiple cortical and subcortical areas, in association with decreased cognitive performance. We identified altered expression of gene pathways involved in PNN regulation, potentially contributing to decreases of PNNs in SZ, including genes involved in CS synthesis and CS chain modification, PNN key components as well as several endogenous proteases that cleave key PNN components. Altered expression of genes involved in glial cell development, axonal conductance at the nodes of Ranvier and myelination indicate a contribution to disrupted glial cell maturation and deficits in myelination and axonal conductance in SZ. Taken together, these results are consistent with growing evidence for widespread ECM abnormalities in multiple brain regions in SZ, contribute to emerging evidence for large‐scale ECM dysregulation in SZ, expand on the molecules involved in ECM abnormalities and brain regions affected and point to molecular pathways involved in PNN decreases, glial cell dysfunction and cognitive impairment in SZ.

## CONFLICTS OF INTEREST

The authors have no conflict of interest to disclose. TK has received consulting income from Alkermes Inc.

## AUTHORS CONTRIBUTIONS

Drs. Haroutunian and Katsel carried out the gene expression studies, statistical analyses, graphic data representation and contributed significantly to manuscript preparation. Drs. Pantazopoulos, Chelini and Klengel contributed to data analyses and manuscript preparation as well immunocytochemistry data on human tissue. Dr. Berretta was a primary contributor to the manuscript preparation.

### PEER REVIEW

The peer review history for this article is available at https://publons.com/publon/10.1111/ejn.15009.

## Supporting information

Table S1‐S6

## Data Availability

The data that support the findings of this study are available from the authors upon reasonable request.
