# Supplementary material for: Molecular signature of extracellular matrix pathology in schizophrenia
Source: Eur J Neurosci. 2020 Nov 13;53(12):3960–87. doi: 10.1111/ejn.15009 (PMC8359380; doi:10.1111/ejn.15009)
Supplement: Supplementary file 1 — Table S1‐S6 [file EJN-53-3960-s001.pdf]

Supplemental Material

Table S1. Interregional ECM-related gene expression differences are diminished in SZ cerebral cortex (as shown on Figure 2).

	UC			SZ		
Gene Symbol	$F_{4,185}$	Sig.*	p(BH)[1]	$F_{4,185}$	Sig.*	p(BH)[1]
SEMA6D	7.35	1.6E-05	4.1E-03	7.10	2.2E-05	1.9E-03
BCAN	6.06	1.3E-03	0.004	3.64	0.007	0.009
CHST1	4.71	0.001	0.014	5.36	3.9E-03	0.0048
SPOCK3	4.41	0.002	0.011	2.97	0.021	0.041
HAPLN2	3.62	0.007	0.015	0.59	ns	ns
SEMA4G	3.28	0.013	0.042	1.95	ns	ns
ADAM10	3.21	0.014	ns	1.50	ns	ns
SPOCK1	2.76	0.029	ns	6.17	0.0001	ns
CHST3	2.55	0.041	ns	0.44	ns	ns
APP	2.39	0.052	ns	2.62	0.036	ns

*One-way ANOVA. [1]Multiple-comparison post-hoc Benjamin-Hochberg (BH) procedure. ns= Non-significant.

Table S2. Differentially expressed ECM genes in cognitively normal controls (CDR0) and individuals with mild cognitive impairment (CDRs 0.5 and 1) across cortical regions only (ANCOVAs using age, sex, PMI and brain pH as covariates).

Gene Symbol	$F_{2,185}$	Sig.*
CD44	18.82	4.60E-17
SRGN	15.37	1.20E-08
IGFBP3	13.35	1.50E-12
DSE	10.89	2.20E-10
CHST3	8.49	3.70E-08
CHSY1[1]		<0.0001
CHST1[1]		<0.0001
ADAMTS1[1]		<0.0001
EGFR[1]		<0.0001
CSPG5	4.88	0.0001
APP	4.51	0.0003
NCAN	4.41	0.0003
SPOCK1	4.2	0.001
BCAN	3.53	0.0025
LHX2[1]		0.005
SPOCK3	2.86	0.0171
IL6ST[1]		0.025

* ANCOVA corrected for Age, sex, PMI and brain pH
[1]For these measures, the nonparametric Kruskal-Wallis procedure was applied.

Table S3. Differentially expressed ECM genes as a function of CDRs (1-5) in SZ across cortical regions only (ANCOVAs using age, sex, PMI and brain pH as covariates).

Gene Symbol	$F_{4,181}$	Sig.*
EGFR	14.59	7.14E-15
CHSY1	11.19	1.17E-11
LHX2	10.72	3.48E-11
IL6ST	10.63	4.21E-11
SRGN	9.22	1.12E-09
CSPG5	7.43	8.23E-08
BCAN	6.23	1.61E-06
CD44[1]		<0.0001
CHST3[1]		<0.0001
NCAN[1]		<0.0001
CHST1[1]		<0.0001
IGFBP3[1]		<0.0001
DSE	4.47	0.0001
SPOCK1	2.67	0.0119
SPOCK3	2.64	0.0128

* ANCOVA corrected for Age, sex, PMI and brain pH

[1]For these measures, the nonparametric Kruskal-Wallis procedure was applied.

Table S4. Sex effect on ECM-related DEGs in SZ across all of the studied regions.

Gene name	Gene Symbol	p (Corr) (SZ)	p (Corr) (SZ-Sex)	p (Corr) (Sex)
CD44 molecule	CD44	**1.82E-07**	**0.007**	0.814
dermatan sulfate epimerase	DSE	**1.34E-07**	**0.016**	0.246
ADAM metallopeptidase with thrombospondin type 1 motif, 1	ADAMTS1	**7.27E-09**	**0.041**	0.255
syndecan 3	SDC3	**0.0035**	0.159	**9.29E-10**
ADAM metallopeptidase domain 2	ADAM2	**2.14E-06**	0.159	**0.026**
epidermal growth factor receptor	EGFR	**0.0002**	0.819	**0.033**
amyloid beta (A4) precursor protein	APP	**0.0015**	0.999	**0.009**

Table S5. TaqMan gene expression assays used in the study

Symbol	Gene name	Assay ID	NCBI accession	Exon boundary
NCAN	neurocan	Hs00189270_m1	NM_004386.2	8-9
BCAN	brevican	Hs01016696_m1	NM_021948.4	2-3
CD44	CD44 molecule	Hs01075864_m1	NM_000610.3	3-4
SRGN	serglycin	Hs01004159_m1	NM_001321053.1	3-4
GUSB	glucuronidase, beta	Hs00939627_m1	NM_000181.3	8–9
RPLP0	ribosomal protein, large, P0	Hs99999902_m1	NM_053275.3	3–3
PPIA	peptidylprolyl isomerase A	Hs99999904_m1	NM_021130.3	4–4

Table S6. Data from published RNAseq and protein expression studies. Listed below are the ECM-related dysregulated genes detected in our analyses (see Figs. 4, 5, 6). Highlighted in blue are genes significantly altered (at genome wide significance level) in the CommonMind_DLPFC dataset

Gene_Symbol	CommonMind_DLPFC (Hoffman et al., 2019)	DISC1_RNA (Wen et al., 2014)	DISC1_Protein (Wen et al., 2014)
**ADAM10**	FC = -1.01 LogFC = -0.02 p = 0.07	FC = -1.22 LogFC = -0.28 p = 0.13	FC = -1.39 LogFC = -0.47 **p <0.005**
**ADAM11**	FC = -1.02 LogFC = -0.02 p = 0.08	FC = 1.58 LogFC = 0.66 **p = 0.05**	NA
**ADAM2**	NA	NA	NA
**ADAM7**	NA	FC = -2.07 LogFC = -1.05 p = 0.32	NA
**ADAM9**	FC = -1 LogFC = -0.01 p = 0.61	FC = -3.04 LogFC = -1.6 **p <0.005**	NA
**ADAMTS1**	FC = -1.05 LogFC = -0.08 p = 0.09	FC = -15.06 LogFC = -3.91 **p <0.005**	FC = -2.77 LogFC = -1.47 **p <0.005**
**ADAMTS12**	NA	FC = -15.94 LogFC = -3.99 **p <0.005**	NA
**ADAMTS16**	FC = 1.01 LogFC = 0.02 p = 0.61	FC = -7.91 LogFC = -2.98 **p <0.005**	NA
**ADAMTS2**	FC = 1.13 LogFC = 0.17 **p <0.005**	FC = -2.05 LogFC = -1.04 p = 0.06	NA
**ADAMTS5**	FC = 1.02 LogFC = 0.04 p = 0.24	FC = -1.25 LogFC = -0.32 p = 0.47	NA
**ADAMTS7**	NA	FC = -1.5 LogFC = -0.59 **p = 0.05**	NA
**ADAMTS9**	FC = -1.04 LogFC = -0.05 p = 0.18	FC = -9.19 LogFC = -3.2 **p <0.005**	NA
**APP**	FC = -1.01 LogFC = -0.01 p = 0.43	FC = -1.42 LogFC = -0.5 p = 0.06	NA
**BCAN**	FC = 1.02 LogFC = 0.04 p = 0.11	FC = 32.38 LogFC = 5.02 **p <0.005**	FC = 1.71 LogFC = 0.78 p = 0.09
**BMP4**	FC = -1.09 LogFC = -0.12 **p = 0.03**	FC = -10.08 LogFC = -3.33 **p <0.005**	NA
**BMP6**	FC = 1.01 LogFC = 0.01 p = 0.78	NA	NA
**BMP7**	FC = 1.02 LogFC = 0.03 p = 0.14	FC = -2.15 LogFC = -1.11 **p <0.005**	FC = -1.81 LogFC = -0.86 p = 0.01
**BMPR1A**	FC = -1.01 LogFC = -0.01 p = 0.32	FC = -1.05 LogFC = -0.07 p = 0.78	NA
**BMPR1B**	FC = -1.01 LogFC = -0.01 p = 0.66	FC = -2.7 LogFC = -1.43 **p = 0.01**	NA
**CD44**	FC = -1.01 LogFC = -0.01 p = 0.85	FC = -3.24 LogFC = -1.7 **p <0.005**	NA
**CHST1**	FC = 1 LogFC = 0 p = 0.83	FC = 2.05 LogFC = 1.03 **p = 0.02**	NA
**CHST3**	FC = 1 LogFC = 0.01 p = 0.83	FC = -1.66 LogFC = -0.74 p = 0.06	FC = -1.33 LogFC = -0.42 **p = 0.04**

**CHSY1**	FC = -1 LogFC = 0<br>p = 0.8	FC = -1.13 LogFC = -0.18<br>p = 0.41	FC = -1.45 LogFC = -0.53<br>**p <0.005**
**CNTF**	FC = -1.02 LogFC = -0.03<br>p = 0.55	NA	NA
**CSPG5**	FC = 1.02 LogFC = 0.02<br>p = 0.21	FC = 2.44 LogFC = 1.29<br>**p <0.005**	NA
**DCN**	FC = 1.01 LogFC = 0.01<br>p = 0.7	FC = -1.7 LogFC = -0.76<br>p = 0.27	FC = -4.8 LogFC = -2.26<br>**p <0.005**
**DSE**	FC = 1.02 LogFC = 0.03<br>p = 0.1	FC = -2.8 LogFC = -1.48<br>**p <0.005**	NA
**EGFR**	FC = -1.03 LogFC = -0.04<br>p = 0.21	FC = 1.03 LogFC = 0.05<br>p = 0.85	NA
**FGF2**	FC = -1 LogFC = -0.01<br>p = 0.8	FC = -2.26 LogFC = -1.17<br>**p = 0.03**	NA
**FGFR1**	FC = 1.04 LogFC = 0.06<br>**p <0.005**	FC = -1.59 LogFC = -0.67<br>**p = 0.01**	NA
**HABP4**	FC = 1 LogFC = 0<br>p = 0.79	FC = -1.17 LogFC = -0.23<br>p = 0.31	NA
**HAPLN1**	FC = 1.02 LogFC = 0.03<br>p = 0.22	NA	NA
**HAPLN2**	FC = -1.04 LogFC = -0.05<br>p = 0.12	FC = 3.44 LogFC = 1.78<br>**p = 0.01**	NA
**HS2ST1**	FC = 1 LogFC = 0.01<br>p = 0.61	FC = -1.76 LogFC = -0.82<br>**p <0.005**	NA
**HS3ST1**	FC = -1.06 LogFC = -0.08<br>p <0.005	NA	NA
**HS6ST1**	FC = -1.01 LogFC = -0.02<br>p = 0.23	FC = -1.17 LogFC = -0.22<br>p = 0.54	FC = 1.52 LogFC = 0.6<br>**p <0.005**
**IGF1**	FC = 1.12 LogFC = 0.16<br>**p <0.005**	NA	NA
**IGF1R**	FC = -1.02 LogFC = -0.02<br>**p = 0.04**	FC = -1.39 LogFC = -0.47<br>**p = 0.02**	NA
**IGFBP3**	FC = -1.02 LogFC = -0.02<br>p = 0.55	FC = -1.09 LogFC = -0.12<br>p = 0.87	NA
**IL6ST**	FC = -1 LogFC = 0<br>p = 0.77	FC = -1.63 LogFC = -0.71<br>**p = 0.01**	FC = -1.42 LogFC = -0.5<br>**p = 0.01**
**LHX2**	FC = 1.03 LogFC = 0.05<br>**p = 0.01**	FC = 3.26 LogFC = 1.7<br>**p <0.005**	NA
**LHX6**	FC = 1.05 LogFC = 0.07<br>**p <0.005**	FC = 15.85 LogFC = 3.99<br>**p <0.005**	NA
**LIFR**	FC = -1.02 LogFC = -0.03<br>**p = 0.05**	FC = -1.97 LogFC = -0.98<br>**p <0.005**	FC = 1.26 LogFC = 0.33<br>**p = 0.01**
**LRP1**	FC = -1.04 LogFC = -0.06<br>**p <0.005**	FC = -1.01 LogFC = -0.01<br>p = 0.97	NA
**MMP16**	FC = -1.02 LogFC = -0.02<br>p = 0.09	FC = 1.42 LogFC = 0.51<br>p = 0.09	NA
**MMP19**	NA	NA	NA
**MMP24**	FC = 1.01 LogFC = 0.02<br>p = 0.23	FC = 1.84 LogFC = 0.88<br>**p = 0.01**	FC = 1.62 LogFC = 0.7<br>**p = 0.04**
**NCAN**	FC = -1.06 LogFC = -0.09<br>**p <0.005**	FC = 4.12 LogFC = 2.04<br>**p <0.005**	NA
**SDC2**	FC = 1.04 LogFC = 0.06	FC = -5.13 LogFC = -2.36	FC = -3.18 LogFC = -1.67

	**p <0.005**	**p <0.005**	**p <0.005**
**SEMA4A**	FC = -1.01 LogFC = -0.01<br>p = 0.58	FC = 1.94 LogFC = 0.96<br>**p = 0.01**	NA
**SEMA4G**	FC = -1 LogFC = -0.01<br>p = 0.79	FC = 1.01 LogFC = 0.01<br>p = 0.98	NA
**SEMA5A**	FC = 1.02 LogFC = 0.02<br>p = 0.28	FC = 2.44 LogFC = 1.29<br>**p <0.005**	NA
**SEMA6A**	FC = 1 LogFC = 0<br>p = 0.88	FC = -2.72 LogFC = -1.44<br>**p <0.005**	FC = -1.9 LogFC = -0.93<br>**p = 0.01**
**SEMA6D**	FC = -1.02 LogFC = -0.03<br>**p = 0.03**	FC = -1.07 LogFC = -0.09<br>p = 0.63	NA
**SPOCK1**	FC = 1 LogFC = 0<br>p = 0.89	FC = 1.27 LogFC = 0.34<br>p = 0.25	NA
**SPOCK3**	FC = -1 LogFC = -0.01<br>p = 0.72	FC = 1.68 LogFC = 0.74<br>p = 0.1	NA
**SRGN**	FC = -1.05 LogFC = -0.06<br>p = 0.08	NA	NA
**TGFBR1**	FC = 1.02 LogFC = 0.03<br>p = 0.21	FC = -1.54 LogFC = -0.62<br>**p = 0.05**	NA
**TGFBR3**	FC = -1 LogFC = -0.01<br>p = 0.81	FC = -5.14 LogFC = -2.36<br>**p <0.005**	NA
